# Hydrogen sulfide in precision medicine: connecting basic science, sensing, and clinical application

**DOI:** 10.4103/mgr.MEDGASRES-D-25-00190

**Published:** 2026-01-06

**Authors:** Shuo Liu, Yao Li, Yingqian Zhang, Xingran Wang, Linhan Zhu

**Affiliations:** 1 Hebei Key Laboratory of Advanced Laser Technology and Equipment, Center for Advanced Laser Technology, Hebei University of Technology, Tianjin, China; 2 Departments of Respiration III, Hebei Children’s Hospital, Shijiazhuang, Hebei Province, China; 3 Department of Pediatrics, Beijing Friendship Hospital, Capital Medical University, Beijing, China

**Keywords:** biomarker, cancer, gasotransmitter, hydrogen sulfide, metabolic disease, neurological, non-invasive diagnosis, QEPAS, respiratory, trace gases, vascular

## Abstract

**Facts**

1. Hydrogen sulfide (H_2_S) is a key endogenous signaling molecule involved in cardiovascular, neurological, respiratory, and cancer-related pathophysiology.
2. Dynamic monitoring of trace H_2_S levels provides critical insight into disease initiation, progression, and therapeutic response.
3. Emerging spectroscopic techniques, particularly quartz-enhanced photoacoustic spectroscopy (QEPAS), offer unprecedented sensitivity and selectivity for H_2_S detection in biological samples.
4. Breath and skin gas analysis represent promising non-invasive approaches for translating H_2_S sensing from bench to bedside.

**Open questions**

1. What are the precise physiological thresholds of H_2_S concentration that differentiate healthy and pathological states across diverse organ systems?
2. How can QEPAS and other spectroscopic methods be miniaturized and standardized for reliable, real-time clinical use?
3. Can H_2_S modulation be safely targeted in therapy without disturbing other gasotransmitter pathways (nitric oxide and carbon monoxide)?
4. What regulatory and validation frameworks are required to translate H_2_S-based diagnostics into routine clinical practice?

Hydrogen sulfide, as a critical endogenous gasotransmitter, plays a significant role in the pathophysiology of various diseases, including cardiovascular diseases, neurodegenerative disorders, respiratory dysfunctions, and cancer. The dynamic fluctuations in hydrogen sulfide concentration are closely associated with the onset and progression of these conditions. The precise monitoring of trace amounts of hydrogen sulfide in biological samples holds substantial value for elucidating disease mechanisms, developing diagnostic biomarkers, and enabling targeted therapies. This review provides a comprehensive overview of the current advancements in hydrogen sulfide detection technologies in medical applications, with a particular focus on the groundbreaking potential of novel spectroscopic techniques in medical diagnostics. This paper through an integrated research approach that bridges hydrogen sulfide biology, sensing technology, and clinical applications—particularly by utilizing breath and skin gas analysis as windows into metabolic and disease states. A core innovation presented in this work is the proposed optimization of the hydrogen sulfide-quartz-enhanced photoacoustic spectroscopy technique for medical settings, which provides a transformative tool for non-invasive disease screening, precise detection, and targeted therapeutic research. The ultimate goal is to bridge the gap between basic research, sensor technology, and clinical needs, driving innovative applications of hydrogen sulfide in precision medicine.

## Introduction

Hydrogen sulfide (H_2_S) was once regarded as a toxic gas but has now been recognized as a crucial endogenous gasotransmitter in mammals, alongside nitric oxide (NO) and carbon monoxide (CO), forming the core members of the gas signaling molecule family.^1^ It is synthesized through enzymatic pathways in the body and plays a central role in cardiovascular homeostasis, neuroregulation, anti-inflammatory responses, and metabolic balance. The physiological and pathological effects of H_2_S exhibit strict concentration dependence and tissue specificity, and disease states can significantly disrupt H_2_S metabolism.^2^ Therefore, precise detection of trace, dynamic levels of H_2_S in complex biological samples is essential for elucidating its pathological mechanisms, developing diagnostic biomarkers, and enabling targeted therapies. This article systematically reviews the current H_2_S detection technologies in medicine and analyzes the principles of various methods and their applications in medical contexts.^3^ Notably, trace gas analysis is demonstrating revolutionary potential in non-invasive medical diagnostics. Exhaled breath and skin-released gases contain rich metabolic and pathological information, providing an attractive “non-invasive window” for early disease screening and monitoring. As a key endogenous/exogenous gas molecule, H_2_S concentration fluctuations in these samples are closely associated with various disease states. Thus, developing high-performance H_2_S detection platforms suitable for these complex sample matrices is a critical step in advancing gas-based medical diagnostics toward clinical applications. In this context, quartz-enhanced photoacoustic spectroscopy (QEPAS) technology, with its ultra-high sensitivity, excellent selectivity, rapid response, and portability, has emerged as an ideal solution to overcome the bottleneck in medical H_2_S detection. By precisely detecting photoacoustic signals using a quartz tuning fork (QTF), QEPAS is particularly well-suited for trace-level analysis of biological gaseous samples with complex backgrounds. At the conclusion of this review, an innovative, clinically translatable H_2_S-QEPAS detection solution is proposed. This solution addresses key medical application challenges by integrating and optimizing gas flow interface design, adopting adaptive background suppression algorithms, and standardizing analytical workflows, with the goal of providing reliable tools for non-invasive disease screening, dynamic efficacy assessment, and mechanistic research. This review comprehensively evaluates the current status and medical applicability challenges of H_2_S detection technologies, the bidirectional regulatory mechanisms and diagnostic/therapeutic potential of H_2_S in vascular, neurological, respiratory, and metabolic diseases, the frontier progress of trace gas analysis (breath/skin-released) in medical diagnostics, the breakthroughs of QEPAS technology in H_2_S detection, and the design of a clinically oriented H_2_S-QEPAS solution. This review aims to promote the precise detection of H_2_S in medical applications.

This work systematically integrates the molecular pathological mechanisms of H_2_S, the prospects for non-invasive gas sample diagnostics, and photoacoustic sensing technology, establishing a cross-disciplinary review framework spanning fundamental research to clinical applications. Not only does it comprehensively evaluate the medical suitability of existing detection methods, but it also innovatively proposes and meticulously designs a clinically translatable QEPAS-H_2_S detection protocol. This protocol integrates optical path engineering with algorithmic denoising, directly addressing the core bottlenecks in current bio-gas detection concerning sensitivity, interference resistance, and operational complexity. It provides a clear technical pathway and theoretical foundation for advancing the practical application of H_2_S as a gas biomarker in precision medicine.

## Methods

Through a comprehensive literature review, we systematically collated all research pertaining to the role of H_2_S in precision medicine, encompassing its fundamental biological properties, detection techniques, and clinical applications. The search scope covered publications up to March 2025, utilizing databases including PubMed, Web of Science Core Collection, and Scopus. Keyword combinations with Boolean operators were employed to maximize coverage. Core search terms comprised (“H_2_S sensor” OR “hydrogen sulphide detection” OR “sensing”) and (“therapy” OR “clinical application” OR “H_2_S disease mechanism”). Specific search syntax was adapted according to each database’s requirements.

Studies were screened according to the following criteria: Inclusion criteria: (1) Original research articles and review papers; (2) Focusing on the physiological/pathological effects of H_2_S, H_2_S sensor development, or clinical trials involving H_2_S; (3) English-language publications with full-text availability. Exclusion criteria: (1) Conference abstracts, editorials, and non-peer-reviewed publications; (2) Literature where H_2_S was not the core research focus.

Literature retrieved through title and abstract screening was subsequently subjected to full-text evaluation to ensure compliance with inclusion criteria. Relevance assessment was conducted on retrieved articles. A total of 52 documents were excluded due to non-compliance in titles or abstracts, leaving 96 for subsequent analysis. Furthermore, two additional documents deemed irrelevant to the study focus were excluded following full-text review. Ultimately, 94 documents were included in the analysis. All 94 articles underwent comprehensive full-text review and analysis. Each publication was meticulously examined to extract research pertaining to H_2_S’s role in precision medicine, encompassing its fundamental biological properties, detection techniques, and clinical applications.

## Status of H_2_S Gas Detection Technology

H_2_S, as an endogenous gas signaling molecule, plays a critical role in the physiological and pathological processes, and its precise monitoring at various concentrations is essential for disease mechanism analysis and optimizing therapeutic strategies. Current H_2_S detection technologies in the medical field primarily rely on electrical, chemical, and spectroscopic sensing principles, each addressing the unique challenges posed by trace, dynamic target analytes in complex biological matrices.^3^ This chapter provides a comprehensive review of the advancements in detection methods suitable for medical applications and discusses their specific usage scenarios in medical diagnostics.

The electrical sensor method detects H_2_S through the interaction between the gas and the electrical properties of sensitive materials, such as current, resistance, or ionic flow. The commonly used sensitive sulfur electrode method offers a broad measurement range, as well as good stability and repeatability, making it widely applied in the determination of endogenous H_2_S concentrations in rat and human plasma. The principle is that H_2_S dissolves in an antioxidant solution and undergoes pH-dependent dissociation, generating S^2−^ under alkaline conditions (pH 10–12),^4^,^5^ which are subsequently detected by the silver/sulfur electrode in conjunction with a reference electrode.^6^ H_2_S concentration is then calculated based on the standard curve.

Chemical sensor detection methods include chemical reagent methods and chemical instrumentation analysis techniques. The methylene blue method is one of the simplest approaches, utilizing the reaction of H_2_S with chemical reagents in an acidic environment to form methylene blue, with H_2_S quantified through absorbance measurements.^7^ However, it exhibits relatively low sensitivity and is only suitable for concentrations in the μM range. Gas chromatography-sulfur chemiluminescence coupling separates H_2_S from other sulfur compounds using gas chromatography, and the sulfur chemiluminescence detector analyzes H_2_S in biological samples.^8^,^9^ This method offers low detection limits and high sensitivity, suitable for the nM range, but requires maintaining the equipment’s airtightness and a long equilibrium time. It is widely used for the determination of exogenous H_2_S. The methyl bromide high-performance liquid chromatography method reacts with H_2_S to generate a specific labeled product, ethylborane sulfide, which is then analyzed by fluorescence and mass spectrometry coupling.^10^ This technique offers higher sensitivity and selectivity, making it suitable for H_2_S detection in disrupted cell and tissue samples.

In recent years, spectroscopy-based detection technologies have garnered increasing attention in the field of H_2_S sensing due to their superior performance in trace gas analysis. These methods, characterized by high sensitivity, excellent selectivity, and outstanding spectral resolution, exhibit significant potential for real-time detection.^11^ Traditional fluorescence probe techniques and emerging spectroscopic approaches have both contributed to this advancement. To date, over a hundred structurally novel, highly sensitive, and real-time fluorescent probes for H_2_S detection have been reported. In recent years, to address the need for highly sensitive and real-time monitoring of H_2_S in biological systems, researchers have developed a variety of fluorescent probes based on specific chemical reactions, which can be broadly classified into four categories according to their reaction mechanisms: nucleophilic substitution, reduction, sulfur cleavage, and metal displacement.^12^,^13^ In the field of nucleophilic substitution strategies, the small-molecule ratio-based photoacoustic probe CyCl-1, based on chlorinated anthocyanin dyes, when the probe is exposed to H_2_S, H_2_S undergoes a nucleophilic substitution reaction with the active chlorine on the probe molecule, causing the probe to produce a double-peak ratio photoacoustic signal change in the near-infrared region. This probe enables high-resolution ratio photoacoustic real-time imaging and quantitative detection of H_2_S in live mice.^14^ The coumarin-NBD probe has demonstrated its rapid and highly selective imaging capability for H_2_S in live cells and zebrafish models.^15^ For reduced probes, their high selectivity has been extended to live cell two-photon imaging and visualization of endogenous H_2_S in cardiac tissue. The probe specifically reduces the nitro group on the nitroalkene moiety of H_2_S to an amino group, significantly weakening the intramolecular charge transfer effect of the coumarin molecule. This causes a blue shift in the absorption spectrum and a significant blue shift in the fluorescence spectrum, along with a dramatic change in the intensity ratio, thereby enabling the detection of H_2_S.^16^,^17^ Subsequently, two studies reported two bay-functionalized trisubstituted porphyrin diamides (PDI 1 and PDI 2), which function as ratio-type near-infrared “switch-on” chemical dosimeters, enabling the differentiation and detection of H_2_S/cysteine in live cells.^18^ A representative example of a sulfur-cleaving probe is the design of a disulfide bond-based probe conjugated with 7-ethyl-10-hydroxycamptothecin (SN-38) as the anticancer drug, proposed mechanism involved disulfide bond cleavage triggered by H_2_S. After disulfide bond cleavage, the fragments underwent intramolecular cyclization, releasing the five-membered ring compound 1,2-dithiolan-3-one, the naphthylamide fluorophore, and the anticancer drug SN-38. This system can be specifically activated by H_2_S in HCT116 cells and mouse tumors, thereby enabling imaging-guided synergistic chemotherapy.^19^ Regarding metal-exchange probes, various Cu(II) coordination probes have been demonstrated to effectively image exogenous H_2_S in live cells, mouse liver tissue, and even whole animals.^20^ Notably, the 64Cu-diacetyl-bis(N_4_-methylthiosemicarbazone)-fluorescein 5-isothiocyanate probe established a positron emission tomography/optical dual-modality in situ tracing platform for H_2_S in the brain and revealed a significant increase in H_2_S levels in the lesion area in an lipopolysaccharide-induced neuroinflammatory model.^21^ In the latest research, an excited state intramolecular proton transfer probe based on 1,3,4-thiadiazole/coumarin provides a new tool for real-time detection of Cu^2+^/H_2_S in food quality control and biomedical research. Its detection mechanism is the nitrogen atom in 1,3,4-thiadiazole and carbonyl oxygen atom on coumarin ring form a hole, which easy to accommodate Cu^2+^. A series of reactions ultimately enables the detection of Cu^2+^/H_2_S.^22^

In parallel, emerging spectroscopic techniques, such as tunable diode laser absorption spectroscopy and QEPAS, have been extensively utilized for the ultra-sensitive detection of gaseous H_2_S.^11^ Tunable diode laser absorption spectroscopy employs precise modulation of the laser wavelength to target the characteristic absorption peaks of H_2_S. Based on the Beer–Lambert law, it interprets attenuation signals to determine gas concentration, offering rapid response and operational simplicity. With advancements such as the incorporation of multi-pass optical cells, its detection sensitivity has been significantly improved to sub-ppm levels.^23^,^24^ QEPAS, leveraging the photoacoustic effect, overcomes limitations of traditional spectroscopy.^25^,^26^ Its signal intensity scales directly with light source power and does not require wavelength scanning. Through innovative acoustic resonator designs, such as gold-coated cells and Helmholtz-type configurations, QEPAS has achieved detection limits in the ppb range. These breakthroughs in photoacoustic sensing lay a robust foundation for the development of interference-resistant platforms for *in vivo* H_2_S detection, thereby advancing the clinical application of gasotransmitters in non-invasive disease diagnostics.

In summary, various H_2_S detection technologies demonstrate significant application potential owing to their distinct working principles and respective advantages. These techniques offer complementary strengths in terms of sensitivity, selectivity, and suitability for different analytical contexts. **Table 1** compares the significant differences between H_2_S detection technologies in terms of key indicators such as detection limit, response speed, and result reproducibility (measured by relative standard deviation), providing empirical evidence for technology selection in specific application scenarios.

**Table 1:** Comparison and analysis of H_2_S detection technologies

Category	Detection range	Response time	RSD	Selectivity	Typical application scenarios
Electrical sensing^4^,^5^,^6^	μM	1-30 s	< 5%	CO, CH_4_, nh_3_	Industrial safety, personal protection
Chemical sensing^7^,^8^,^9^,^10^	μM-nM	0.5-5 min	< 10%	SO_2_, thiol compounds, amines	Environmental emergency response, biological sample analysis
Fluorescent probe^12^,^13^,^14^,^15^,^16^,^17^,^18^,^19^,^20^,^21^,^22^	μM-nM	10 s-10 min	5-15%	Other nucleophiles, pH sensitivity	Live-cell imaging, in vitro diagnostics
Spectral detection^23^,^24^,^25^,^26^	ppb-ppm	1-5 s	< 1%	Virtually interference-free	Medical breath analysis, environmental monitoring, precision process control

CH_4_: Methane; CO: carbon monoxide; H_2_S: hydrogen sulfide; NH_3_: ammonia; RSD: relative standard deviation; SO_2_: sulfur dioxide.

## Role of H_2_S in Medicine

The endogenous production of H_2_S primarily depends on the activity of three key enzymes: cystathionine β-synthase (CBS), cystathionine γ-lyase (CSE), and 3-mercaptopyruvate sulfurtransferase (3-MST). These enzymes regulate H_2_S biosynthesis in a tissue-specific manner through distinct substrates and catalytic mechanisms, thereby contributing to the diverse physiological functions of H_2_S. CBS is predominantly expressed in the nervous system and accounts for approximately 60% of total H_2_S production in the brain. It plays a critical role in maintaining cognitive function by modulating synaptic plasticity and antioxidant defenses. Genetic knockout of CBS leads to a ~70% reduction in hippocampal H_2_S levels and is associated with impaired learning and memory. CSE is highly expressed in the liver, kidneys, neurons, macrophages, and smooth muscle cells, with the highest expression observed in hepatic tissue. It contributes significantly to systemic H_2_S levels and participates in various physiological processes, including inflammation regulation and vascular tone modulation. 3-MST is predominantly localized in red blood cells and the mitochondria of cardiomyocytes, where it accounts for up to 80% of H_2_S production in erythrocytes. In cardiac tissue, 3-MST cooperates with CSE to maintain H_2_S homeostasis, sustaining intracellular concentrations in the range of approximately 50–100 nM.^27^

Based on the above, H_2_S is a crucial gaseous signaling molecule within cells. Recent studies have elucidated its pivotal role in various physiological processes and its close association with a wide range of diseases.

### H_2_S and vascular diseases

As an endogenous vascular regulatory factor, H_2_S plays a crucial role in maintaining vascular homeostasis. It not only inhibits the abnormal proliferation of vascular smooth muscle cells, but also regulates cell apoptosis and autophagy, while modulating hemodynamics through the control of vascular tone. Research has demonstrated that the downregulation of H_2_S signaling pathways is closely associated with the pathogenesis of various vascular diseases, including hypertension, atherosclerosis, and pulmonary arterial hypertension.^28^

Supplementation with H_2_S has been shown to delay or block the progression of these diseases through multiple mechanisms, a hypothesis that has been validated in animal models, cell-based assays, and clinical studies. For instance, exogenous H_2_S donor sodium hydrosulfide (NaHS) significantly reduced tail arterial pressure in spontaneously hypertensive rats and delayed the transition from prehypertension to sustained hypertension.^29^ In renal vascular and salt-sensitive hypertension models, H_2_S improves endothelial-dependent relaxation function, lowers blood pressure, reverses aortic remodeling, and inhibits the renin-angiotensin-aldosterone system activity. Regarding atherosclerosis, H_2_S deficiency promotes plaque instability, whereas exogenous H_2_S supplementation effectively slows the progression of atherosclerosis in mice by downregulating the expression of the chemokine C-X3-C motif chemokine ligand 1 and its receptor CX3C chemokine receptor 1.^30^ Bibli et al.^31^ found that endogenous H_2_S stabilizes arterial plaques and inhibits lipid deposition in *Apoe* knockout mice, suggesting a potential anti-atherosclerotic effect. In pulmonary arterial hypertension models, CSE expression and activity are significantly reduced, leading to decreased H_2_S synthesis.20 Supplementation with exogenous H_2_S not only restores plasma H_2_S levels but also significantly alleviates vascular remodeling. Clinical studies have also shown that H_2_S supplementation reduces the contraction tension of human arterial rings by 42.3% and lowers pulmonary arterial pressure by approximately 17.73%. Furthermore, abnormal H_2_S metabolic pathways may contribute to the development of ischemic heart disease and left ventricular dysfunction.^32^ Studies have demonstrated that overexpression of CSE or administration of H_2_S donors can improve cardiac function and reduce structural abnormalities, thereby providing theoretical support for the therapeutic application of H_2_S in cardiovascular diseases.

### H_2_S and respiratory diseases

H_2_S exerts multiple biological effects in the respiratory system, including regulation of alveolar epithelial cell function, reduction of oxidative stress, and stabilization of the endothelial barrier. Studies have shown that H_2_S can improve mitochondrial function in alveolar epithelial cells via a sirtuin 1 (SIRT1)-dependent mechanism, reducing oxidative stress and thereby inhibiting cigarette smoke extract-induced cellular senescence and apoptosis.^33^,^34^

Additionally, H_2_S can block cigarette smoke-induced airway remodeling in mice by reversing SIRT1-mediated oxidative stress and epithelial-mesenchymal transition, suggesting a protective role in smoking-related lung diseases.^35^,^36^ H_2_S has also demonstrated strong intervention potential in acute lung injury. Animal experiments indicate that exogenous H_2_S donors (e.g., NaHS) or inhalation of H_2_S gas can effectively alleviate lipopolysaccharide-induced inflammatory responses, reduce the expression of inflammatory cytokines such as interleukin-6 and tumor necrosis factor-α, and improve the pathological state of lung tissue.^37^ In pneumonia, clinical findings reveal a negative correlation between serum H_2_S levels and high-sensitivity C-reactive protein, suggesting that H_2_S could serve as a potential biomarker for infection severity.^38^ Furthermore, H_2_S can modulate viral infections by inhibiting the replication of respiratory syncytial virus and paramyxoviruses, mitigating virus-induced cellular damage and inflammatory responses, thus holding potential for antiviral therapy.^39^ However, high concentrations of H_2_S may exacerbate the condition by interacting with mitochondrial cytochrome oxidases, interfering with the oxidative phosphorylation process of the respiratory chain, leading to energy metabolism disruption and cellular apoptosis. Additionally, H_2_S may promote bacterial resistance by interfering with immune pathways and cytokine generation. Certain pathogens escape antibiotic effects by inhibiting H_2_S-metabolizing enzymes, thereby increasing the risk of antimicrobial resistance. In tuberculosis, host-generated H_2_S has been shown to enhance the metabolic activity of *Mycobacterium tuberculosis*, accelerating pathogen growth and suppressing immune responses, which contributes to the development of drug resistance.^40^,^41^ In asthma patients during acute exacerbation, sputum H_2_S levels increase while serum concentrations decrease, reflecting the dynamic role of H_2_S in airway inflammation and reactivity.^42^ For chronic obstructive pulmonary disease, smoking is a major risk factor, and it is noteworthy that serum H_2_S levels are significantly lower in smokers compared to non-smokers.^43^ NaHS, as an H_2_S donor, can inhibit cigarette smoke-induced inflammation, injury, and apoptosis of alveolar epithelial cells in a mouse model of chronic obstructive pulmonary disease through the prolyl hydroxylase 2/hypoxia-inducible factor-1α/mitogen-activated protein kinase signaling pathway, thereby alleviating chronic bronchitis induced by cigarette smoke and improving lung function. Moreover, low levels of H_2_S treatment can effectively improve lung function and reduce tissue pathological changes, pulmonary edema, and permeability through inhibition of the transforming growth factor-β1 pathway, further mitigating cigarette smoke-induced chronic obstructive pulmonary disease.^35^,^44^,^45^ These findings highlight the potential therapeutic role of H_2_S in patients with chronic obstructive pulmonary disease. Additionally, the levels of H_2_S in exhaled breath can predict the inflammatory phenotype in patients with chronic obstructive pulmonary disease, suggesting its potential value in disease classification and personalized treatment. In conclusion, H_2_S exhibits a wide and complex range of mechanisms in respiratory system diseases.

### H_2_S and neurological disorders

In the central nervous system, H_2_S is synthesized through the catalytic actions of CBS and 3-MST and is distributed across neurons, astrocytes, and microglia, where it regulates various cellular processes.^46^,^47^,^48^ Research has demonstrated that H_2_S exerts significant neuroprotective effects in the central nervous system, primarily through alleviating oxidative stress, modulating synaptic plasticity, maintaining mitochondrial homeostasis, and regulating cell death.^49^ At the molecular level, H_2_S improves mitochondrial function by activating the SIRT1/peroxisome proliferator-activated receptor γ coactivator-1α axis, enhances the nuclear factor erythropoietin-2-related factor 2/heme oxygenase-1 antioxidant pathway,^50^,^51^ and increases the capacity of cells to eliminate reactive oxygen species and reactive nitrogen species.^52^,^53^ Additionally, H_2_S regulates the Warburg effect, promoting aerobic glycolysis to maintain neuronal metabolic activity, thereby improving cell survival under energy deficiency or toxic stress.^54^ H_2_S also modulates autophagy and apoptosis, inhibiting mitochondrial-mediated apoptosis, reducing endoplasmic reticulum stress, and stabilizing autophagic flux, which significantly mitigates cell damage induced by neurotoxic factors such as homocysteine, methyl-4-phenylpyridinium (MPP^+^), and amyloid-β.^55^,^56^ The role of H_2_S in synaptic function is particularly prominent, as it enhances long-term potentiation in the hippocampus, increases the channel activity of N-methyl-D-aspartic acid and α-amino-3-hydroxy-5-methyl-4-isoxazole-propionic acid receptors, stabilizes synaptic structures, and improves synaptic transmission efficiency, thus enhancing learning and memory abilities.

Investigation showed that plasma H_2_S levels in patients with depression are negatively correlated with the severity of the depression, suggesting that a reduction in H_2_S may contribute to the pathogenesis of depression. In Alzheimer’s disease, impaired H_2_S production is considered a key factor in disease onset, with Alzheimer’s disease patients exhibiting significantly lower plasma and brain H_2_S levels compared to healthy individuals. The exogenous H_2_S donor NaHS has been shown to improve spatial learning and memory in Alzheimer’s disease mouse models, indicating its potential as an anti-Alzheimer’s disease therapy.^57^ Parkinson’s disease, a common neurodegenerative disorder, is associated with the inhibition of endogenous H_2_S synthesis by MPP^+^.^58^ However, exogenous H_2_S has been shown to counteract MPP^+^-induced neuronal apoptosis and stress responses, demonstrating its potential in combating Parkinson’s disease. In conclusion, H_2_S, as a gaseous neuromodulator, plays a crucial role in maintaining neurovascular homeostasis and shows promising potential as a therapeutic agent for neurological diseases.

### H_2_S and cancer and metabolic diseases

H_2_S, a molecule with dual regulatory potential, exhibits a concentration-dependent “bell-shaped” effect in metabolism-associated tumors, such as hepatocellular carcinoma. At low concentrations, H_2_S promotes tumorigenesis, whereas high concentrations inhibit tumor growth. The enzymes CBS and CSE, which are involved in H_2_S biosynthesis, are highly expressed in the liver, suggesting that H_2_S plays a critical role in both physiological and pathological processes within this organ. In the pathogenesis of hepatocellular carcinoma, low concentrations of H_2_S activate pro-oncogenic signaling pathways, including nuclear factor-κB, epidermal growth factor receptor/extracellular signal-regulated kinase/matrix metalloproteinase-2, thereby enhancing cancer cell survival, proliferation, and migration. Studies have demonstrated that NaHS treatment significantly enhances nuclear factor-κB activation, increases anti-apoptotic capacity, and promotes invasiveness in liver cancer cells.^59^ Conversely, high concentrations of H_2_S exhibit notable anti-cancer effects, with exogenous NaHS significantly inhibiting liver cancer cell proliferation, inducing autophagy and apoptosis, and restricting tumor migration and invasion.^60^,^61^ Furthermore, H_2_S interacts with other gaseous signaling molecules, such as CO and NO, and plays a crucial role in regulating tumor activity. For instance, treatment with H_2_S, CO, or NO donors in human breast cancer cells can inhibit tumor progression by modulating reactive oxygen species tolerance, anti-proliferative capacity, and pro-apoptotic responses.^62^ In metabolic diseases, H_2_S has been shown to mitigate the progression of non-alcoholic fatty liver disease and non-alcoholic steatohepatitis by regulating lipid metabolism, suppressing inflammation, and improving mitochondrial function. These metabolic disorders are significant contributors to the onset of liver cancer.^63^,^64^ Thus, H_2_S serves as both a bridge and a catalyst between metabolic diseases and tumorigenesis.

In conclusion, H_2_S, as a gaseous signaling molecule, plays a pivotal role in the pathophysiology of hepatocellular carcinoma and metabolic-related diseases. At low concentrations, it facilitates tumor progression, while at high concentrations, it exerts anti-tumor effects. This “dual regulation” model not only underscores the complexity of H_2_S in liver cancer pathophysiology but also provides a theoretical foundation for its potential targeted application in cancer therapy. Future research into the regulatory mechanisms of H_2_S synthesis, the cross-talk of signaling pathways, and the dose-dependent effects of H_2_S will facilitate breakthroughs in the precision treatment of metabolic tumors, including liver cancer.

**Table 2** summarizes the main targets of H_2_S in various disease systems, the main molecular mechanisms, trends in disease manifestation, and treatment directions. H_2_S regulates multiple signaling pathways and participates in a wide range of physiological processes, playing crucial roles in cardiovascular, respiratory, neurological, and metabolic diseases, as well as in cancer. Relevant therapeutic strategies include the development of H_2_S donors, regulation of key enzyme expression, and combination with existing drugs, all of which demonstrate promising translational prospects.

**Table 2:** Role of H_2_S in different kinds of diseases

Disease	Functional target	Main molecular mechanism	H_2_S trend	Treatment direction
Cardiovascular diseases^28^,^29^,^30^,^31^,^32^	RAAS, CX3CL1/CX3CR1	Maintenance of vascular homeostasis, immune regulation, antioxidant	Decline	H_2_S donor, CSE activator combined with RAAS blockade
Respiratory diseases^33^,^34^,^35^,^36^,^37^,^38^,^39^,^40^,^41^,^42^,^43^,^44^,^45^	SIRT1, PHD2/HIF-1α, TGF-β1, MAPK	Anti-fibrosis, epithelial barrier protection, inflammation relief	Decline	Inhalation of sustained-release H_2_S, signal pathway regulation
Neurological diseases^46^,^47^,^48^,^49^,^50^,^51^,^52^,^53^,^54^,^55^,^56^,^57^,^58^	SIRT1/PGC-1α, Nrf2/HO-1, NMDA/AMPA	Neuroprotection, synaptic plasticity regulation, mitochondrial homeostasis	Decline	CBS/3-MST activation, neuroprotective agent
Cancer and metabolic diseases^59^,^60^,^61^,^62^,^63^,^64^	NF-kB, EGFR/ERK, MMP-2/-9	Concentration-dependent tumor regulation, metabolic reprogramming	Colorectal cancer show upregulation of H_2_S; other decline	Inhibition or activation of H_2_S synthase, combined metabolic intervention

3-MST: 3-Mercaptopyruvate sulfurtransferase; AMPA: α-amino-3-hydroxy-5-methyl-4-isoxazole-propionic acid; CBS: cystathionine β-synthase; CSE: cystathionine γ-lyase; CX3CL1: C-X3-C motif chemokine ligand 1; CX3CR1: CX3C chemokine receptor 1; EGFR: epidermal growth factor receptor; ERK: extracellular signal-regulated kinase; H_2_S: hydrogen sulfide; HIF-1α: hypoxia-inducible factor-1α; HO-1: heme oxygenase-1; MAPK: mitogen-activated protein kinase; MMP: matrix metalloproteinase; NF-kB: nuclear factor-KB; NMDA: N-methyl-D-aspartate; Nrf2: nuclear factor erythropoietin-2-related factor 2; PHD2: prolyl hydroxylase 2; RAAS: renin-angiotensin-aldosterone system; SIRT1: sirtuin 1; TGF-β1: transforming growth factor-β1.

## H_2_S is a Trace Gas in Medical Testing

In the previous section, we discussed in detail the critical role of H_2_S gas in medicine. Although the concentration of H_2_S in the human body is relatively low, typically in the ppm to ppb range, it is a trace gas that plays an essential role in both physiological and pathological processes. Due to its low concentration, the study of H_2_S and other similar gases as potential biomarkers has garnered increasing attention in the medical field. This chapter will focus on the application of trace gases in medical diagnostics, covering their detection in respiratory gases and skin-released gases.

### Breathing gas detection

In disease diagnosis, the direct observation of pathological changes within the human body remains limited by current imaging or sampling methods. In contrast, analyzing trace components in exhaled gases allows for non-invasive, real-time monitoring of affected areas and the progression of diseases.^65^ Compared to traditional blood or urine tests, breath analysis offers significant advantages in terms of ease of sampling, smaller sample requirements, and processing efficiency.^66^ However, in the approximately 500 mL of exhaled air, only about 350 mL may carry volatile organic compounds related to diseases, with the remainder serving a dilutive role.^67^ As a result, the concentrations of biomarkers available for analysis are typically in the ppb to ppm range, presenting high demands for sensitivity and selectivity in detection technologies.

As shown in **Table 3**, various gases have been identified as potential biomarkers in exhaled breath in current research, including 13C isotopes, NO, CO, NH_3_, acetone, H_2_, and others.^68^,^69^,^70^,^71^,^72^,^73^,^74^,^75^,^76^ The combination of these biomarkers forms a “breath fingerprint,” which is driving the early diagnostic determination of diseases.^77^

**Table 3:** Potential biomarkers in exhaled breath

Marker gas	Associated diseases	Gas source	Detection limit	Clinical application
^13^CO_2_^69^	*Helicobacter pylori* infection	Gastric bacterial metabolites	< 1% (isotope ratio)	Non-invasive diagnosis, suitable for all age groups
NO^70^	Asthma, bronchitis	Signaling molecules produced by airway epithelial cells	20 ppb	Inflammation grading and precise asthma management
CO^71^	Neonatal jaundice, oxidative stress	Hemoglobin metabolism and oxidative stress products	26 ppb	Newborn screening and oxidative stress assessment
H2^72^,^73^,^74^	Intestinal flora activity	Small intestinal bacterial fermentation products	8.86 ppm	Assessment of digestive function and microbial flora status
nh_3_^75^	Liver/kidney dysfunction	Amino acid degradation and liver and kidney metabolism	14 ppb	Monitoring of liver/kidney function and assessment of disease progression
Acetone^76^	Asthma monitoring	Fatty acid metabolism	6.85 ppb	Asthma classification and blood glucose control monitoring

^13^CO_2_: Carbon-13 labeled carbon dioxide; CO: carbon monoxide; H_2_: hydrogen; NH_3_: ammonia; NO: nitric oxide.

Research in the field of breath gas analysis began with the use of 13C isotopes for detecting *Helicobacter pylori* and NO for diagnosing asthma.^69^ As early diagnosis of respiratory, metabolic diseases, and renal and hepatic dysfunctions has gained increasing attention, exhaled gas analysis has emerged as an important non-invasive diagnostic tool. Significant progress has been made in the diagnostic applications of breath gas analysis for various diseases in recent years, providing new technical support for the early diagnosis and treatment of related conditions.

In respiratory diseases, NO is widely used as a biomarker for conditions such as asthma, bronchiectasis, and rhinitis. Grossel et al.^70^ achieved a detection limit of approximately 20 ppb for NO using a quantum cascade laser and Helmholtz resonance photoacoustic cell. Additionally, the concentration of CO is closely related to the diagnosis of neonatal jaundice. Li et al.^71^ based on the principle of photoacoustic heterodyne, a breath sensor for non-invasive detection of CO in human exhaled air has been developed, with a detection limit of 26 ppb. The measurement of H_2_ concentration is also used in the diagnosis of lactose intolerance and small intestinal bacterial overgrowth.^74^ Shrestha et al.^73^ utilized the portable breath analyzer breath analyzer to measure H_2_, while Mao et al.^72^ indirectly measured H_2_ concentration by determining the photoacoustic cell resonance frequency shift, achieving a detection limit of 8.86 ppm. In renal and hepatic diseases, elevated NH_3_ concentration is commonly associated with dysfunction. Shang et al.^75^ developed a QEPAS human exhaled NH_3_ detection system with a detection limit of 14 ppb, successfully monitoring exhaled NH_3_ levels in healthy volunteers. In metabolic diseases, acetone concentration is closely related to diabetes. A study used an Nd-YAG pulsed laser to detect acetone, with a sensitivity and specificity of 89% and 93%, respectively, effectively distinguishing between healthy volunteers and diabetic patients.^76^ Acetone is also associated with asthma, and photoacoustic technology can effectively differentiate asthma patients from the normal population.^78^ These studies indicate that breath gas detection methods play a critical role in the early diagnosis of various diseases, providing reliable support for clinical practice. In summary, the application of trace gases in exhaled gas analysis involves interdisciplinary fields such as laser and optical engineering, physics, chemistry, and medicine, requiring further cross-collaboration to address the key technical challenges faced in practical clinical applications.

### Skin release gas detection

The monitoring of O_2_ and carbon dioxide (CO_2_) concentrations is essential for the assessment of respiratory function and metabolic status, and is of particular clinical importance in the management of acute conditions and chronic diseases. In this context, techniques for the detection of skin-released gases have been progressively developed as an important non-invasive monitoring tool.

The beginnings of skin gas monitoring technology date back to the 1970s, with the first breakthroughs coming from electrochemical sensors, in particular the Clark and Severinghaus electrodes, which were first used for O_2_ detection, measuring the concentration of O_2_ in a liquid by means of an electrochemical reaction.^79^ This technology enabled O_2_ monitoring and was gradually extended to clinical applications such as neonatal intensive care, while the Severinghaus electrode was used for CO_2_ measurement, which reflected the CO_2_ concentration through a chemical reaction on the surface of the electrode, pioneering a new approach to CO_2_ monitoring.^80^ In the 21^st^ century, with the development of technology, optical sensors emerged as a new generation of gas monitoring technology. Optical sensors work on the principle of fluorescence burst, which means that the concentration of a gas is deduced by measuring the change in light intensity of a fluorescent material. A significant advantage of optical sensors over electrochemical sensors is that they do not consume O_2_, enabling stable monitoring over long periods of time. In particular, fluorescence lifetime sensors avoid errors caused by external factors, such as changes in light intensity and movement of the sensor position, by measuring changes in the lifetime of fluorescent molecules, thus improving the accuracy and stability of monitoring.^81^,^82^ In addition, these optical sensors are gradually being integrated into wearable devices, making continuous monitoring possible, especially in athletic, chronically ill, or other individuals who require long-term tracking. As optical technology continues to advance, electron paramagnetic resonance sensors have become a new research direction. Electron paramagnetic resonance sensors utilise the paramagnetic properties of O_2_ to derive the concentration of O_2_ by measuring the interaction of O_2_ molecules with a probe. This technique is not only highly sensitive to low concentrations of O_2_, but also exhibits high stability and accuracy over long periods of time, making it particularly suitable for applications that involve long-term monitoring of the skin.

**Figure 1:**
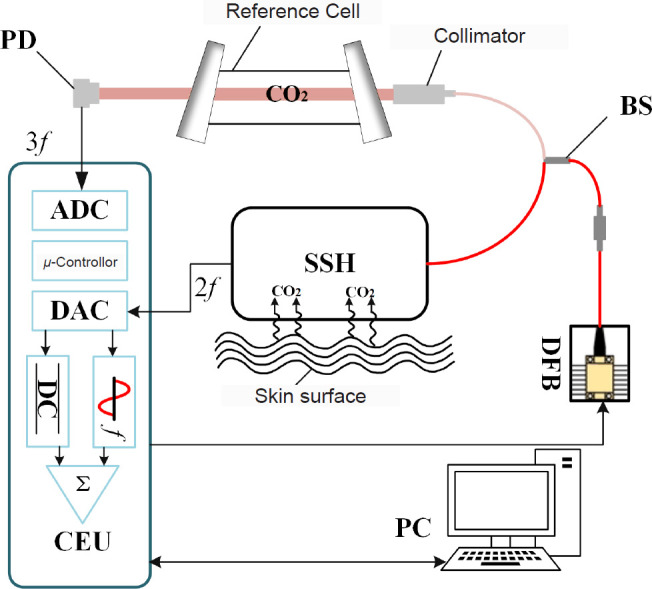
Schematic diagram of the skin breathing sensor. Created with Microsoft Visio Drawing (2021 edition). ADC: Analog-to-digital converter; BS: blood sensor; CO_2_: carbon dioxide; CEU: central processing unit; DAC: digital-to-analog converter; DC: direct current; DFB: distributed feedback laser; PC: personal computer; PD: photodiode; SSH: signal shaping & amplification.

For the detection of CO_2_, optical techniques are also used, especially in sensors based on fluorescence burst and fluorescence lifetime measurements. These sensors reflect changes in gas concentration through the interaction of CO_2_ with a fluorescent dye in a way that avoids the temperature dependence and calibration problems associated with conventional electrochemical techniques. A number of fluorescent dye-based CO_2_ sensors have been successful in achieving high sensitivity and fast response, and are particularly useful in clinical settings for patients requiring real-time monitoring. Optical thin film and fiber optic sensors are also being developed for gas monitoring as technology continues to innovate. Optical thin-film sensors are capable of providing fast response to O_2_ and CO_2_ with long stability, especially in wearable devices, and can provide comfortable and long-term gas monitoring. In addition, sensors based on new technologies such as electron paramagnetic resonance and photoacoustic spectroscopy also show good application prospects, and in the past 2 years of research, a CO_2_ detection device based on photoacoustic spectroscopy has been gradually developed,^83^,^84^ and its detection principle is shown in **Figure 1**, which realises continuous monitoring of CO_2_ release from the skin of the human small arm, and this technology can provide high-precision gas monitoring, and it does not require heating equipment, which improves the portability and applicability of the device.^85^ A recent study proposed a contactless wearable epidermal flux sensor that constructs a closed microcavity on the skin surface and integrates wireless sensors for real-time quantitative monitoring of gases such as water vapour, volatile organic compounds and CO_2_ on the skin surface, and the results showed that the device is capable of sensitively assessing the status of wound healing, skin barrier integrity and infections, which is of significant potential application value for clinical care.^86^ Overall, skin gas detection technology has undergone continuous development from the initial electrochemical sensors to optical sensors to modern wearable devices. Advances in each generation of technology have not only improved the accuracy and stability of gas detection, but have also enabled these technologies to be more widely used in clinical monitoring, personalised health management, and telemedicine, and are expected to provide real-time, accurate health monitoring for more patients in the future.

## Quartz-enhanced Photoacoustic Spectroscopy and Medical H_2_S Detection

Optical sensing technology based on spectroscopy utilizes the absorption characteristics of gas molecules with good selectivity and high sensitivity. In particular, gas sensing devices developed based on QEPAS have become a hot spot in current research due to the advantages of high sensitivity and good real-time performance. It has been widely used in the fields of atmospheric environment monitoring, industrial on-site detection and medical aid diagnosis.

### Application of QEPAS in trace H_2_S detection

The QEPAS technology has demonstrated excellent performance in the field of trace gas detection and has become an important direction in the development of high-sensitivity sensors. In the studies exploring the improvement of the detection performance of QEPAS systems, the main approaches are to increase the excitation intensity by using high-power lasers and to optimize the QTF structure so as to improve the energy accumulation time. In the study of Ma et al.,^87^ a QEPAS system was constructed for CO detection by using a high-power laser with a wavelength of 4.61 μm as the excitation light source, and successfully achieved a very low detection limit of 1.5 ppb very low detection limit. Subsequently, Duquesnoy et al.^88^ designed a novel QTF with a wide fork-finger gap and combined it with a cylindrical acoustic resonator cavity, which was applied to a QEPAS-based CO_2_ detection system, and achieved a detection limit of 9 ppb in the 1.5 μm band. Subsequently, Lin et al.^89^ developed a QTF with a resonance frequency of 28 kHz and used it in conjunction with a miniature acoustic resonator to significantly improve the system performance, ultimately achieving a minimum detection limit of 325 ppb for gases. In a recent study, Ma et al.^90^ proposed a novel conical QTF, which successfully improved the signal-to-noise ratio of the system by a factor of 3.02 and reduced the minimum detection limit of the target gas to 16.45 ppb. These results fully validate the excellent sensitivity of the QEPAS technology for trace gas detection, and indicate that the technology has a promising future in demanding applications such as medical diagnosis.

In the continuous development of QEPAS technology, the optimization of detection for specific target gases has gradually become a research focus. Among them, H_2_S, as a biomarker related to many diseases, is particularly attractive for its trace detection in medical diagnosis. In order to achieve high sensitivity, fast response and low interference detection of H_2_S, researchers have not only continued to innovate the sensor structure, but also systematically optimized key components such as acoustic wave enhancement module, laser source configuration and signal processing methods.^26^ Early research focused on the near-infrared band, focusing on increasing the laser power. By introducing an erbium-doped fiber amplifier, researchers have increased the laser power to 1.4 W. This breakthrough has greatly improved the sensitivity of the near-infrared QEPAS, allowing the detection limit to reach 142 ppb.^91^ Although the absorption coefficient in the near-infrared band is relatively low, the power enhancement via the erbium-doped fiber amplifier enabled to gain a relative advantage in cost. On this basis, subsequent research has focused on further improving the sensitivity by optimizing the generation of optoacoustic signals, specifically in the design of the QTF, which has a direct impact on the performance of the QEPAS system. The researchers have enhanced the acoustic signal acquisition by improving the design of the QTF with a large spacing, which enables the system to detect signals more accurately at lower gas concentrations.^88^,^89^,^90^ In addition, by optimizing the structural design of the QTFs, the noise interference was reduced, which further enhanced the stability and sensitivity of the system. Further, the silicon cantilever beam fiber optic sensor technology proposed by Guo et al.^92^ achieved a detection limit of 10.96 ppb for H_2_S in an SF_6_ background, demonstrating the great potential of the QEPAS technology in real-world environments. In their latest study, Meng et al.^93^ proposed a miniaturized platform with a T-type absorber chamber with a volume of only 154.34 μL, which achieved an ultra-low detection limit of 86.1 ppb of H_2_S within a 1000-second integration time, which is 2–3 orders of magnitude smaller than the conventional multichannel system, and is suitable for marker in wearable or portable medical diagnostic devices for the system is suitable for the analysis of markers in wearable or portable medical diagnostic devices.

In summary, QEPAS technology continues to evolve through near-infrared laser power compensation, QTF design improvement, validation of anti-jamming performance in complex backgrounds, and construction of miniaturized detection platforms. It not only improves the detection sensitivity, but also enhances the adaptability and expandability of the system in complex environments, providing a solid theoretical and engineering foundation for the medical applications of H_2_S in breath analysis, metabolism monitoring, and early disease warning. In the future, QEPAS technology is expected to play a greater role in personalized medicine and non-invasive diagnosis.

### H_2_S-QEPAS system in medicine

**Figure 2:**
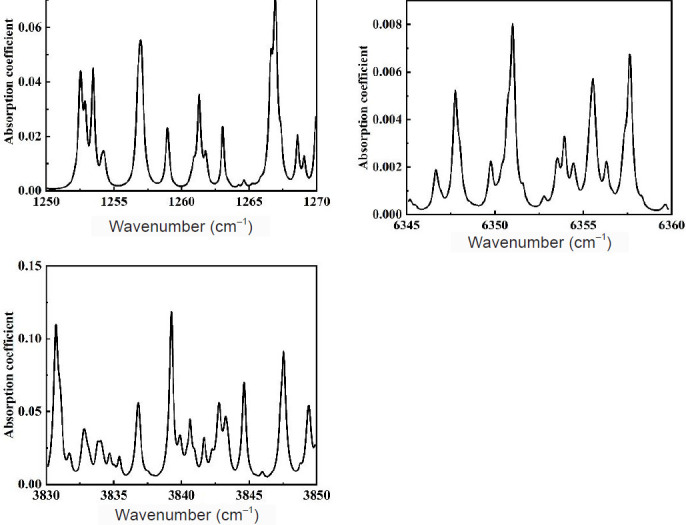
H_2_S absorption spectrum The absorption spectra of the H_2_S gas in these three wavelengths from the HITRAN database (https://hitran.org/).^94^ H_2_S: Hydrogen sulfide.

QEPAS technology, as a highly sensitive and non-contact trace gas detection method, is gradually attracting attention in medical detection. Regarding the design of a QEPAS system program for the detection of H_2_S in medicine, it is crucial to select the appropriate gas absorption spectra firstly, the absorption region of H_2_S gas spectra is mainly concentrated in the near-infrared wavelengths of 1.58 and 2.6 μm, and the mid-infrared wavelengths of 7.8 μm. **Figure 2** shows the absorption spectra of the H_2_S gas in these three wavelengths from the HITRAN database (https://hitran.org/).^94^ From this, the wavelength range of the laser for the H_2_S detection system can be determined. In practical applications, considering the cost problem, the laser with a center wavelength of 1574 nm was selected as the excitation light source for most experiments, with a wavelength tuning range of 1570–1580 nm and an output laser power of about 15 mW.

**Figure 3:**
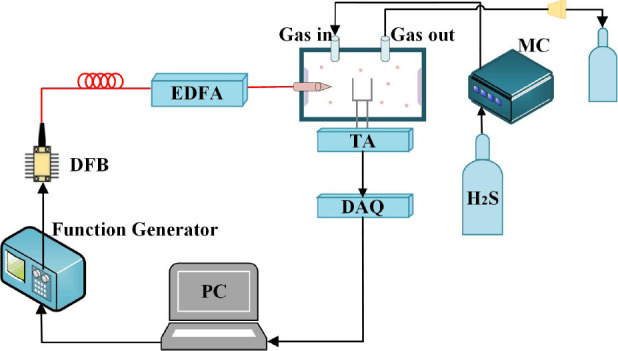
Medical H_2_S-QEPAS detection system Created with Microsoft Visio Drawing (2021 edition). DAQ: Data acquisition system; DFB: distributed feedback laser; EDFA: erbium-doped fiber amplifier; H_2_S: hydrogen sulfide; MC: microphone cell; PC: personal computer; QEPAS: quartz-enhanced photoacoustic spectroscopy; TA: tuning fork amplifier.

The selection of acoustic wave detector element as the core device for photoacoustic signal detection is very important for gas sensing system. In the QEPAS system, the acoustic wave detector adopts piezoelectric crystal QTF resonator, which has the advantages of small size, low price, low power consumption, and extremely insensitive to environmental noise, and it is a widely used electronic component. Secondly, in order to achieve high sensitivity detection of H_2_S gas in medicine, it is also necessary to optimize the design of the structure of the gas chamber, and according to the finite element software simulation, to design a suitable gas chamber to be fused and installed with the acoustic wave detector, to obtain the largest possible detection signal. Since the signal converted by the QTF through the piezoelectric effect is an extremely weak current pulse, it needs to be amplified and converted by a preamplifier. The processed signal is then transferred to the computer via the data acquisition card, and finally, the harmonic signals are demodulated using a lock-in amplifier to calculate the gas concentration to be measured. The overall detection diagram of the integrated design system is shown in **Figure 3**.

## Limitations

This review has several limitations that should be considered. First, the discussion of H_2_S-pathology correlations is largely based on preclinical and *in vitro* studies; further large-scale human clinical validation is needed to confirm these mechanisms and their diagnostic applicability. Second, although the proposed H_2_S-QEPAS system offers a promising detection strategy, its actual performance in real clinical environments—such as under varying patient physiological conditions or in the presence of complex gas mixtures—requires further extensive testing. In addition, this review may be constrained by the scope of literature included; although efforts were made to include key studies, the rapidly evolving nature of this field means that some very recent technological advances may not be covered. These limitations highlight the need for more clinical-translational studies and technical validation in real-world settings.

## Conclusion

This review systematically elucidates the core regulatory mechanisms of H_2_S as a key gas transmitter in cardiovascular, neurological, respiratory, and metabolic diseases, highlighting its significant value for early diagnosis and targeted therapy of diseases; it also demonstrates the unique advantages of QEPAS technology in the medical detection of trace H_2_S — its ppb-level sensitivity, It also demonstrates the unique advantages of QEPAS technology in trace H_2_S medical detection—its ppb-level sensitivity, anti-interference ability and portability provide an innovative platform for non-invasive diagnosis. The H_2_S-QEPAS optimization scheme proposed in this study overcomes the challenges of humidity interference and complex matrix in biological sample detection by integrating a low-flow clinical sampling interface, an adaptive background noise suppression algorithm and a standardized operating procedure, which significantly improves the reliability of clinical applications. This solution opens up a new path for real-time monitoring of respiratory diseases, dynamic assessment of neurodegenerative lesions and metabolic studies of cancer. This review not only summarizes past progress but also proposes a technical framework directly applicable for clinical validation. Future research should focus on: conducting large-scale clinical trials, establishing disease-specific H_2_S concentration reference values, and advancing the miniaturization and integration of sensing devices. These critical steps will propel gas signaling molecule detection from laboratory research towards precision medicine practice, ultimately realizing the application potential of H_2_S as a key biomarker in modern healthcare.

## Data Availability

*Not applicable*.
